# The prevalence of insufficient iodine intake in pregnancy in Africa: protocol for a systematic review and meta-analysis

**DOI:** 10.1186/s13643-019-1092-7

**Published:** 2019-08-22

**Authors:** Charles Bitamazire Businge, Benjamin Longo-Mbenza, Andre Pascal Kengne

**Affiliations:** 1 Department of Medicine, Faculty of Health Sciences, University of Cape Town, Cape Town, South Africa; 2 Department of Obstetrics and Gynaecology, Faculty of Health Sciences, Walter Sisulu University, Private Bag x1 WSU, 5117, Mthatha, South Africa; 3 Faculty of Medicine, University of Kinshasa, Kinshasa, Democratic Republic of Congo; 4 Non-Communicable Disease Research Unit, South African Medical Research Council, Cape Town, South Africa

**Keywords:** Iodine, Insufficiency, Pregnancy, Africa

## Abstract

**Background:**

Insufficient iodine intake in pregnancy is associated with many adverse pregnancy outcomes. About 90% of African countries are at risk of iodine deficiency due to poor soils and dietary goitrogens. Pregnancy predisposes to insufficient iodine nutrition secondary to increased physiological demand and increased renal loss. Iodine deficiency is re-emerging in countries thought to be replete with pregnant women being the most affected. This review seeks to identify the degree of iodine nutrition in pregnancy on the entire African continent before and after the implementation of national iodization programmes.

**Methods:**

A systematic search of published literature will be conducted for observational studies that directly determined the prevalence of insufficient iodine intake among pregnant women in Africa. Electronic databases and grey literature will be searched for baseline data before the implementation of population-based iodine supplementation and for follow-up data up to December 2018. Screening of identified articles and data extraction will be conducted independently by two investigators. Risk of bias and methodological quality of the included studies will be assessed using a risk of bias tool. Appropriate meta-analytic techniques will be used to pool prevalence estimates from studies with similar features, overall and by major characteristics including the region of the study, time period (before and after implementation of iodization programmes), sample size and age. Heterogeneity of the estimates across studies will be quantified and publication bias investigated. This protocol is reported according to Preferred Reporting Items for Systematic reviews and Meta-Analysis protocols (PRISMA-P) 2015 guidelines.

**Discussion:**

This review will help ascertain the impact of national iodization programmes on the iodine nutrition status in pregnancy in Africa and advise policy on the necessity for monitoring and mitigating iodine deficiency in pregnancy in Africa. This review is part of a thesis that will be submitted to the Faculty of Health Sciences, University of Cape Town, for the award of a PhD in Medicine whose protocol has been granted ethics approval (UCT HREC 135/2018). In addition, the results will be published in a peer-reviewed journal.

**Systematic review registration:**

PROSPERO CRD42018099434

**Electronic supplementary material:**

The online version of this article (10.1186/s13643-019-1092-7) contains supplementary material, which is available to authorized users.

## Strengths and limitations of this study

- This will be the first systematic review and meta-analysis aiming to estimate the level of iodine deficiency among pregnant women in Africa.
- Methodological and statistical procedures that will be used to derive accurate estimates are robust and reliable.
- This review may be limited by the degree of accuracy of the various methods used to measure urine iodine concentration.
- Since urine iodine concentration varies according to the time day when the sample was collected, this may introduce some degree of heterogeneity
- Further heterogeneity may be introduced by studies with small sample sizes.

## Background

Although iodine deficiency affects over 2 billion people worldwide and is re-emerging in formerly iodine-replete industrialised countries, pregnant women, lactating mothers and their offspring are more susceptible to the adverse effects of iodine deficiency [1]. These include stillbirths, miscarriages, intrauterine growth restriction, postpartum thyroiditis, subclinical and overt hypothyroidism, dyslipidemia, neuro-cognitive and psychomotor deficits [2–4].

In endemic areas, chronic iodine deficiency among women in reproductive age will be exacerbated by the increased renal clearance and loss in urine which will predispose the foetus to defective neuronal migration, myelination and glial differentiation which are key features of brain central nervous system development [4, 5]. This is the underlying cause of cretinism in severe cases and neurocognitive and psychomotor deficits. Hence, whole generations will be at risk of chronic thyroid, metabolic and mental diseases, leading to low socio-economic productivity in areas without sustained adequate nutrition [2].

There is a trend towards the re-emergence of iodine deficiency in iodine-replete countries such as the USA, the UK, New Zealand and Australia [6–9]. This has partially been attributed to inadequate use of iodized salt and voluntary instead of universal iodisation of salt used in commercial and household food production. As a result, the median urine iodine concentration (UIC) in the USA declined from 320 μg/l to 144 μg/l between 1971 and 2010 while pregnancy median UIC fell from 153 μg/l between 2001 and 2006 to insufficient levels <150 μg/l between 2007 and 2010 [6–8]. Data from a survey of 21 European countries in 2014 revealed that despite the iodine status of some countries being adequate across all age groups, 13/21 countries had inadequate iodine intake during pregnancy due to poor access to iodized foodstuffs and inadequate of monitoring of iodine nutrition status [9].

Iodine deficiency is widespread in Africa such that without iodine supplementation, almost 90% of the population will be at risk of iodine deficiency [10–12]. This is mainly due to iodine-deficient soils and goitrogens of which the most significant being poorly detoxified cassava which is rich in thiocyanate [13].

By early 1996, iodine deficiency disorder control programmes using iodised salt as the long-term strategy had been initiated in almost all of the 50 countries in Africa where WHO estimated that iodine deficiency disorder was of public health significance. As a result, more than 50% of the salt consumed in Africa was iodised [14]. Although universal iodisation of salt is the main source of dietary iodine in most African countries, other major sources of dietary iodine in some African countries include groundwater in Somalia and Djibouti and bouillon cubes and canned and processed foods such as in Senegal and Ghana [1, 15–18].

By 2017, 85% of the African countries had achieved sufficient iodine nutrition in the general population [19]. However, only four of the eleven African countries that had median pregnancy UIC survey data (South Africa, Tanzania, Sierra Leone and Ghana) had adequate iodine intake during pregnancy; five (Burkina Faso, Egypt, Niger, Morocco, and Senegal) had insufficient intake during pregnancy, while Djibouti and Liberia had more than enough iodine intake during pregnancy.

Since about 90% of dietary iodine intake is excreted in the urine, the World Health Organization (WHO) recommended that urinary iodine concentration (UIC) is a good marker of recent iodine intake. Hence, median UIC has been used to map out populations at increased risk of thyroid disorders due to iodine deficiency [20]. The median school-age children (SAC) UIC is commonly used to estimate the iodine nutrition status of the most population, but this may underestimate the degree of iodine deficiency in pregnancy due to differing dietary habits of SAC and pregnant women, in addition to specific physiological changes of pregnancy [9, 21, 22].

During pregnancy, the urinary iodine excretion increases by about 30–50% secondary to the increased blood volume, hyperdynamic state and the increased renal blood flow and glomerular filtration [23, 24]. Furthermore, serum iodide concentration is progressively reduced in the second and third trimesters by increased trans-placental transfer to the growing foetus which begins production of thyroid hormones from about 20 weeks’ gestation [25]. In addition, there is extra iodine demand due to the physiological increase in maternal thyroid hormone output. This is as a result of the oestrogen mediated increase in thyroid-binding globulin that progressively decreases the free T4 in the serum, transfer of iodine to the foetus and increased renal iodine clearance [26]. Therefore, women with mild-to-moderate iodine deficiency may develop severe iodine deficiency in pregnancy with resultant subclinical hypothyroidism (SCH), overt hypothyroidism (OH) or isolated T4 deficiency with resultant maternal and offspring short term and long-term complications [25–27].

Hence, the WHO recommends that the average iodine intake to maintain normal thyroid clearance and cater for renal losses in pregnancy should be at least 200 μg daily for pregnant women compared to 100–150 μg per day for non-pregnant women [1]. Among pregnant women, a median UIC < 150, 150–249, 250–499 and > 500 μg is considered an estimate of, respectively, insufficient, adequate, more than adequate and excessive iodine nutritional status [28].

However, there is a paucity of data on the magnitude of iodine deficiency among pregnant women on the continent of Africa [29] and around the globe [19]. We intend to conduct a systematic review and meta-analysis of observational studies carried out to establish the trend in the state of iodine nutrition among pregnant women in Africa following the implementation of national iodization programmes.

## Rationale

Although much gain in access to iodised salt has been achieved in most African countries since the early 1990s, of recent, the implementation of universal salt iodization, the major method of iodine supplementation, seems to be slowing down [7]. Due to challenges with monitoring [30], it is not clear if iodine deficiency may be re-emerging in African countries as in industrialised nations. Like elsewhere around the globe, women in reproductive age, pregnant women and their children will be the most affected, yet the degree of iodine deficiency in pregnancy in Africa is not well documented.

Although the most recent Iodine Global Network (IGN) data suggests that 85% of the African countries have sufficient iodine nutrition in the general population [19], further evaluation of these statistics using the method described by De Benoist et al [30] reveals that 30% of these countries have more than > 50% of the general population with a median UIC < 100 μg/L. This implies a high risk of insufficient iodine intake at the inception of pregnancy among women in reproductive age given that adequate iodine nutrition status in pregnancy is defined by median UIC of 150–249 μg/L [1]. It is recommended that pregnant and lactating women, who make up the most vulnerable portion of the general population, should be considered for supplementation with iodine until the population-based iodization programme is scaled up. Not only is it necessary to achieve sufficient iodine nutrition status, but it is also equally important to sustain adequate iodine nutrition status of the entire population especially the most vulnerable portions. Hence, the impact of iodization should be monitored at a regional or national level at least every 5 years.

## Objectives

The aim of this systematic review and meta-analysis is to ascertain the trend in the prevalence of insufficient iodine nutrition status (median UIC < 150 μg/L) among pregnant women in Africa following the implementation of national iodization programmes and to establish if this has had a sustainable positive impact on the iodine nutrition status of pregnant women in Africa.

## Review questions

The purpose of this review is to address the following questions:

1. What was the prevalence of insufficient iodine intake (UIC < 150 μg/L) among pregnant women on the various African countries before the implementation of national iodine deficiency disorder control programmes?
2. How has the iodine nutrition status during pregnancy changed in the various African countries following the implementation of national iodine deficiency disorder control programmes between 1994 and 31 December 2018?
3. What was the iodine nutrition status of pregnant women in various African countries between 2005 (the year designated by the WHO for the elimination of iodine deficiency through national iodisation programmes [1]) and 2018?

## Methods

### Eligibility criteria

#### Inclusion criteria

The selection of studies for inclusion in the review will be guided by the Population, Intervention/exposure, Comparison and Outcome protocol as stipulated below.

The population comprises of pregnant women on the African continent, the exposure is the period during the implementation of iodine deficiency disorder control programmes from 1994 to 2018, and the comparison is the period before the implementation of iodine deficiency disorder control programmes in 1994. The outcome is the iodine nutrition status during pregnancy in Africa.

Cross-sectional, case-control and cohort studies conducted on iodine deficiency among pregnant women in Africa with data available on mean or median urine iodine concentration will be included in this systematic review. The iodine nutrition status will be defined according to the WHO/ICCIDD classification of iodine intake of populations using median urinary iodine concentration [1]. All studies reported in the English, French or Portuguese languages and conducted on human subjects will be considered.

#### Exclusion criteria

Studies with the following characteristics will be excluded: studies conducted among populations of African origin but residing outside Africa, studies lacking prevalence rates and with the absence of data to compute them, case series with small sample sizes (sample less than 30 participants), and studies not performed in human participants or published in languages other than English, French and Portuguese.

### Source of information

The methods of this systematic review are reported in accordance with the Preferred Reporting Items for Systematic reviews and Meta-Analysis protocols (PRISMA-P) 2015 Guidelines [31] (Additional file 1: Table S1).

### Search strategy for study identification

#### Electronic searches

We will search PubMed-MEDLINE, Google Scholar, SCOPUS, ISI Web of Science (Science Citation Index), Africa Wide Information, African Index Medicus (AIM) and AFROLIB databases for published studies on iodine deficiency in pregnancy in Africa up to 31 December 2018. This search shall be conducted using a predefined comprehensive and sensitive search strategy combining relevant terms with names of countries in Africa, to obtain the maximum possible number of studies. This search will be guided by the African search filter, which has been reported to have good sensitivity (and improved precision) of 74% (1.3–9.4%) and 73% (5–28%) for MEDLINE and EMBASE, respectively [32]. This search filter includes names of each African country and shortened terms to capture studies from regions. Countries with official names in a language other than English will also be entered in the official form, and for countries that have changed names over time, both names shall be included in the search. Table 1 depicts the main search strategy to be employed. We will search reference lists of relevant citations for articles of interest.

**Table 1:** Search strategy for MEDLINE and adaptability to regional databases

Search	Search items	Hits
1	iodine deficiency [tw] OR iodine insufficiency [tw] OR insufficient iodine intake [tw] OR insufficiency iodine nutrition [tw] OR iodine	
2	urine iodine excretion [tw] OR urine iodine concentration [tw] OR urinary iodine excretion [tw] OR urinary iodine concentration [tw] OR urine iodine	
3	Pregnancy [tw] OR Pregnant women [tw] OR expectant mothers [tw] first trimester [tw] [tw] OR second trimester [tw] third trimester [tw]	
4	#1 AND #3	
5	#2 AND #3	
6	African filter((((Angola[tw] OR Benin[tw] OR Botswana[tw] OR “Burkina Faso”[tw] OR Burundi[tw] OR Cameroon[tw] OR “Cape Verde”[tw] OR “Central African Republic”[tw] OR Chad[tw] OR Comoros[tw] OR Congo[tw] OR “Democratic Republic of Congo”[tw] OR Djibouti[tw] OR “Equatorial Guinea”[tw] OR Eritrea[tw] OR Ethiopia[tw] OR Gabon[tw] OR Gambia[tw] OR Ghana[tw] OR Guinea[tw] OR “Guinea Bissau”[tw] OR “Ivory Coast”[tw] OR “Cote d’Ivoire”[tw] OR Kenya[tw] OR Lesotho[tw] OR Liberia[tw] OR Madagascar[tw] OR Malawi[tw] OR Mali[tw] OR Mauritania[tw] OR Mauritius[tw] OR Mozambique[tw] OR Namibia[tw] OR Niger [tw] OR Nigeria[tw] OR Principe[tw] OR Reunion[tw] OR Rwanda[tw] OR “Sao Tome”[tw] OR Senegal[tw] OR Seychelles[tw] OR “Sierra Leone”[tw] OR Somalia[tw] OR “South Africa”[tw] OR Sudan[tw] OR Swaziland[tw] OR Tanzania[tw] OR Togo[tw] OR Uganda[tw] OR “Western Sahara”[tw] OR Zambia[tw] OR Zimbabwe[tw] OR “Central Africa”[tw] OR “Central African”[tw] OR “West Africa”[tw] OR “West African”[tw] OR “Western Africa”[tw] OR “Western African”[tw] OR “East Africa”[tw] OR “East African”[tw] OR “Eastern Africa”[tw] OR “Eastern African”[tw] OR “South African”[tw] OR “Southern Africa”[tw] OR “Southern African”[tw] OR “sub Saharan Africa”[tw] OR “sub Saharan African”[tw] OR “subSaharan Africa”[tw] OR “subSaharan African”[tw] NOT “guinea pig” [tw] NOT “guinea pigs” [tw] NOT “aspergillus niger” [tw]))))	
7	# 4 AND # 6 Limits: 01/01/1990 to 31/12/2018 in English, French, and Portuguese on humans	
8	# 5 AND # 6 Limits: 0109/1990 to 31/12/2018 in English, French, and Portuguese on humans

**Table 2:** Risk of bias assessment tool

	Risk of bias item	Yes = 1 No = 0
External validity		
1	Was the study target population a close representation of the national population in relation to relevant variables?	
2	Was the sampling frame a true or close representation of the target population?	
3	Was some form of random selection used to select the sample, OR, was a census undertaken?	
4	Was the likelihood of non-participation bias minimal?	
Internal validity		
5	Were data collected directly from the participants (as opposed to medical records)?	
6	Were acceptable case definitions of iodine deficiency in pregnancy used?	
7	Were reliable and accepted diagnostic methods for iodine intake utilised?	
8	Was the same mode of data collection used for all participants?	
9	Were the numerator(s) and denominator(s) for the calculation of the iodine intake appropriate?	
Summary of the overall risk of bias	Low risk	0–3
	Intermediate risk	4–6
	High risk	7–9

#### Grey literature

We will search for national ministries of health, international organisations such as the WHO, UNICEF, ICCIDD and IGN, other non-government organisations’ reports, conference and workshop proceedings using Google Scholar search engine and major relevant websites such as WHO African Index Medicus and African Journals Online (AJOL). Key experts in the field will be contacted for any unpublished study.

### Study records

#### Data management

All identified entries will be entered into endnote software for de-duplication of records. Prior to the screening of studies, investigators shall create standardised questions according to the inclusion criteria which will then be pre-tested on a sample of eligible studies.

#### Screening

Two investigators will independently select studies that meet inclusion criteria. Citations and abstracts will be screened for possible inclusion, and duplicate citations will be excluded. Titles and abstracts will then be screened following inclusion criteria described above, following which the full texts of potentially eligible articles will be obtained. These full texts will be screened using a standardised and pre-tested form to include eligible studies. Disagreements will be resolved by consensus or consultation of a third author. Corresponding authors of potentially eligible studies that did not report data that are relevant to our study analysis will be contacted. Reasons for exclusion of non-eligible studies will be documented. The whole selection process will be summarised in a flow chart.

#### Data extraction

Two investigators will independently extract data from included studies, using a standardised and pre-tested data extraction form. Any inconsistencies or disagreement shall be resolved by consensus or consultation with the third investigator.

#### Data items

Data will include the geographic region and country where study was conducted, the year study was carried out and year of publication, the language of publication, demographic characteristics of participants (such as mean age), study design, setting (rural or urban, health-facility or community-based), sample size, and the criteria used for determination of the iodine intake. The median (25th–75th percentiles) and or mean (standard deviation) UIC will be recorded.

#### Assessment of methodological quality and risk of bias

Two reviewers will independently score the quality of included studies. The STROBE checklist [33] will be used to evaluate reporting methodology in each paper while risk of bias in individual studies will be assessed using the risk of bias tool for prevalence studies [34] (Table 2) and the Cochrane guidelines available in Review Manager V.5.3 (http://tech.cochrane.org/revman).

Discrepancies will be resolved by consensus or by consulting the third investigator. Inter-rater agreement on screening, data abstraction and methodological quality will be assessed using Cohen’s κ coefficient [35]. We intend to present the risk of bias and quality scores in a table.

#### Data synthesis, analysis and assessment of heterogeneity

Prevalence data will be summarised by country and country-specific geographic regions where applicable (Table 3). For studies with sufficient data, meta-analysis using random effects models will be conducted overall, that is, across all possible eligible studies. In addition, we will conduct subgroup analysis according to major study-level characteristics such as by country, regions within Africa (as defined by the United Nations); the time period of data collection: before 2005 and after 2005 (the target year for elimination of iodine deficiency through national iodization programmes); the period defined as before and after the implementation of national iodization programmes; and the sample size (below vs. at or above median sample size across included studies) and by age group (below vs. at or above median mean age across included studies). Other criteria for subgroup analyses will include urinary iodine assessment methods and study design. Data will be presented as forest plots showing estimates of mean UIC in pregnancy. For data unsuitable for meta-analysis, we will provide a narrative description of major study characteristics and trends over time.

**Table 3:**
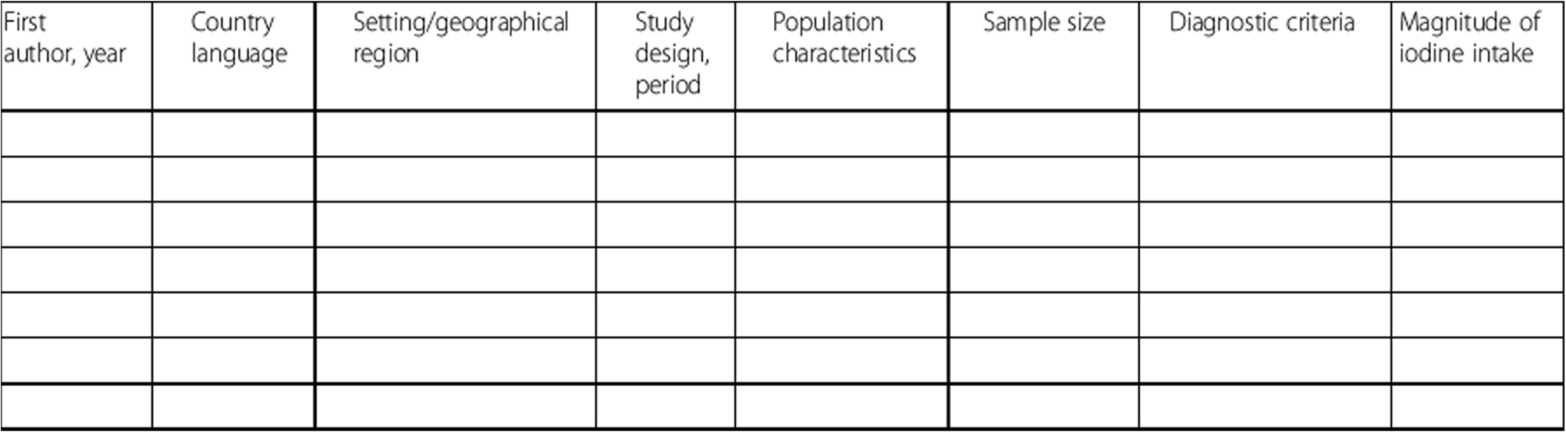
Data synthesis template

Study-specific estimates will be pooled after stabilising the variance of individual studies with the use of Freeman-Tukey double arc-sine transformation [36]. This transformation will help reduce the effect of extremely high or extremely low prevalence rates on the pooled estimate. Heterogeneity will be evaluated by the Cochrane’s *Q* statistic and *I*^2^. *I*^2^ values of 25%, 50% and 75% will respectively be deemed to represent low, medium and high heterogeneity, respectively. Funnel plots together with the Egger test of bias will be used to investigate the publication bias [37].

### Sensitivity analysis

Subgroup analysis using the variables mentioned above and further analysis according to the quality of the studies will be carried out in order to identify possible sources of the heterogeneity. If subgroup differences are identified, they will be described, and the data will be interpreted in light of these differences.

The Duval and Tweedie trim-and-fill will be used to adjust estimates for the effects of potential publication bias. Data analyses will use the ‘*meta*’ package of the statistical software R (version 3.3.3 [2017-03-06], The R Foundation for statistical computing, Vienna, Austria), and the ‘meta’ package.

#### Reporting of this review

The proposed systematic review will be reported following the PRISMA guidelines [38]. We intend to publish a PRISMA checklist alongside the final report.

### Potential amendments

We do not intend to make any amendments to the protocol, to avoid the possibility of outcome reporting bias. However, any amendments that do prove necessary will be documented and reflected online on the PROSPERO website where the protocol has been registered [PROSPERO CRD42018099434].

## Discussion

The degree of iodine nutrition during pregnancy all over the African continent following the implementation of USI and other methods of iodization is not known with certainty. It is not certain whether the trend towards the re-emergence of iodine deficiency among pregnant women in several developed countries around the world is also affecting pregnant women in Africa. A high prevalence of iodine deficiency among pregnant women in Africa would imply an enormous but probably unrecognised predisposition to iodine deficiency disorders affecting not only pregnant women but also lactating mothers and their offspring. The association of iodine deficiency in pregnancy with various adverse pregnancy outcomes and chronic neurocognitive, psychomotor thyroid and cardiovascular diseases among mothers and their offspring requires concerted attention. This review seeks to address the knowledge gap on the magnitude of insufficient iodine intake among pregnant women on the African continent. The data will help shed light on the magnitude of iodine deficiency in pregnancy in Africa which can help inform policy makers on the degree and desirable methods for intervention and the appropriate frequency of monitoring of iodine nutrition status in pregnancy.

Possible limitations of this study would include a predominance of poor quality studies and significant heterogeneity of studies precluding further analysis.

## Additional file

Additional file 1: **Table S1.** PRISMA-P 2015 checklist. (DOCX 30 kb)

## Abbreviations

ICCIDD: International Council for Control of Iodine Deficiency Disorders
IGN: Iodine Global Network
PRISMA-P: Preferred Reporting Items for Systematic reviews and Meta-Analysis protocols
SAC: School-age children
STROBE: Strengthening the Reporting of Observational Studies in Epidemiology
UIC: Urine iodine concentration
WHO: World Health Organization
UNICEF: United Nations Children’s Education Fund
USI: Universal salt iodization
